# Improvement of Surface-Enhanced Raman Scattering Method for Single Bacterial Cell Analysis

**DOI:** 10.3389/fbioe.2020.573777

**Published:** 2020-09-17

**Authors:** Yingchun Yan, Yong Nie, Liyun An, Yue-Qin Tang, Zimu Xu, Xiao-Lei Wu

**Affiliations:** ^1^Institute of New Energy and Low-carbon Technology, Sichuan University, Chengdu, China; ^2^College of Engineering, Peking University, Beijing, China; ^3^Institute of Ocean Research, Peking University, Beijing, China

**Keywords:** Raman microscopy, SERS, bacteria, single cell analysis, bacteria detection

## Abstract

Surface-enhanced Raman scattering (SERS) is a useful tool for label-free analysis of bacteria at the single cell level. However, low reproducibility limits the use of SERS. In this study, for the sake of sensitive and reproducible Raman spectra, we optimized the methods for preparing silver nanoparticles (AgNPs) and depositing AgNPs onto a cell surface. We found that fast dropwise addition of AgNO_3_ into the reductant produced smaller and more stable AgNPs, with an average diameter of 45 ± 4 nm. Compared with that observed after simply mixing the bacterial cells with AgNPs, the SERS signal was significantly improved after centrifugation. To optimize the SERS enhancement method, the centrifugal force, method for preparing AgNPs, concentration of AgNPs, ionic strength of the solution used to suspend the cells, and density of the cells were chosen as impact factors and optimized through orthogonal experiments. Finally, the improved method could generate sensitive and reproducible SERS spectra from single *Escherichia coli* cells, and the SERS signals primarily arose from the cell envelope. We further verified that this optimal method was feasible for the detection of low to 25% incorporation of ^13^C isotopes by the cells and the discrimination of different bacterial species. Our work provides an improved method for generating sensitive and reproducible SERS spectra.

## Introduction

Microbes are the most diverse and abundant organisms on Earth and play crucial roles in biogeochemical carbon and nitrogen cycling. Traditional methods, such as cell culture, have been used to detect and study microorganisms. Recently, culture-independent methods, such as meta-“omics” (MacLean et al., 2009; Scholz et al., 2012), gene chips (He et al., 2007; DeAngelis et al., 2011), and PCR (Aw and Rose, 2012), have been developed and are useful tools for investigating microbes that cannot be cultured in the laboratory. However, these methods are limited in regard to speed, cost, sensitivity, or failure in correlation with the biochemical traits of the target species. In addition, they are not suitable for single-cell analysis, which is important for understanding the biochemical roles of microorganisms in their natural habitats (Davey and Kell, 1996; Amann and Fuchs, 2008; Kalisky and Quake, 2011).

In the last decade, certain tools, including nanometer-scale secondary ion mass spectrometry (NanoSIMS) (Orphan et al., 2001) and a combination of microautoradiography and fluorescence *in situ* hybridization (MAR-FISH) (Kindaichi et al., 2004), have been used to detect the functions and activities of single microbial cells *in situ* within complex communities. Among these tools, Raman spectroscopy combined with microscopy (Raman microspectroscopy) has received increasing attention for label-free analysis of bacteria at the single cell level (Wagner, 2009; Huang et al., 2010; Li et al., 2012b). Due to the high spatial resolution, Raman microspectroscopy can characterize single bacterial cells in a non-invasive and non-destructive manner based on their chemical information, which enables discrimination of different bacterial species at the single cell level (Jarvis and Goodacre, 2004; Berus et al., 2020). This technology can also be combined with stable isotope probing (SIP) (Wang Y. et al., 2016; Wang et al., 2020), FISH (Fernando et al., 2019), and cell sorting, which makes it possible to identify bacterial cells with desired functions from a complex microbial community and even capture the desired cells for subsequent genome sequencing or cultivation (Song et al., 2016; Lee et al., 2019). However, unenhanced Raman spectroscopy is inherently weak, with just one in 10^6^–10^8^ photons incident on the sample, and requires a long acquisition time (Huang et al., 2010). Several efforts have been made to shorten the acquisition time and improve the analytical throughput. For example, a more powerful laser source has been used to conquer the weak scattering signal but might damage biological samples (Kahraman et al., 2008). Recently, resonance Raman spectroscopy has been used to characterize bacteria species at the single cell level. However, this method is often used to detect and screen carotenoid-containing photosynthetic microorganisms (Li et al., 2012a) because it depends on the resonance Raman-active compounds, such as carotenoids.

The surface-enhanced Raman scattering (SERS) effect was first discovered in 1974 (Fleischmann et al., 1974). SERS allows identification of analytes in contact with or very close to plasmonic nanostructures and offers an improvement with enhancement factors of up to 10^6^–10^14^ (Lombardi and Birke, 2009) compared with unenhanced Raman scattering. Like conventional Raman spectroscopy, SERS can be combined with other techniques for specific purposes, such as microspectroscopy, SIP, and single cell sorting (Jarvis and Goodacre, 2004; Li et al., 2012b; Kubryk et al., 2015). It can also be implemented with other modifications of normal Raman scattering, including surface-enhanced resonance Raman scattering (SERRS) (Millo et al., 2011; Bodelon et al., 2016) and tip-enhanced Raman scattering (TERS) (Deckert-Gaudig and Deckert, 2011).

In recent years, due to the high sensitivity, SERS and its modified techniques have been widely used for microbiological research at the single cell level. For example, it has been used to detect bacteria in water (Zhou et al., 2014) and blood (Boardman et al., 2016; Wei et al., 2018; Zhao et al., 2018), discriminate live and dead bacteria (Zhou et al., 2015), and discriminate different bacteria species based on the fingerprint characteristic of SERS (Weiss et al., 2019). Although SERS shows great potential in microbiological research, certain bottlenecks should be addressed. The main drawback of SERS is low reproducibility, to which several factors contribute. First, colloidal silver nanoparticles (AgNPs) are the metal nanoparticles most often used for SERS, where the AgNPs are simply mixed with the bacterial cells (Kahraman et al., 2011). Unstable nanoparticles and poor attachment of nanoparticles to the bacterial cell surface can lead to highly heterogeneous SERS spectra. Second, the microbial SERS signals are mainly attributed to flavins (Zeiri et al., 2004), peptidoglycan (Jarvis and Goodacre, 2004), and adenine-derived compounds (Kubryk et al., 2016). Uncontrollable release of the above compounds induced during sample preparation can result in erroneous spectra. Thirdly, long-time incubation of cells and AgNPs would decrease the reproducibility. Because Raman signals of the target molecules are enhanced when they are very close to metal nanoparticles (Panneerselvam et al., 2018), long-time incubation was usually used to deposit the AgNPs on the cell surface and obtain ideal enhancement. Moreover, although methods for AgNPs preparation have been reported, the effects of the AgNP preparation on SERS are still unclear.

In this work, we optimized the methods used for AgNPs preparation and deposition onto a cell surface. Using *Escherichia coli* as the target, the improved method generated sensitive and reproducible SERS spectra at the single cell level. Different bacterial species and SIP were also implemented to verify the application potential of the improved method for use in microbiological research.

## Materials and Methods

### Preparation of Silver Nanoparticles

**FIGURE 1:**
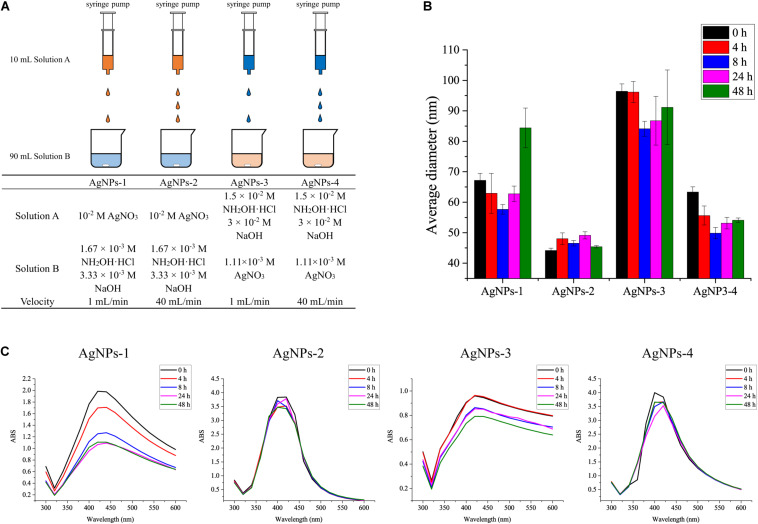
**(A)** Different AgNP preparation methods and **(B)** the average diameters, and **(C)** stability of AgNPs during storage for 48 h at 4°C. Each experiment was replicated three times.

The standard chemicals peptone, yeast extract were purchased from Sigma-Aldrich (Shanghai, China), NaCl, KCl, Na_2_HPO_4_, KH_2_PO_4_, sucrose, silver nitrate and hydroxylamine hydrochloride were purchased from Sinopharm (Shanghai, China). ^13^C_6_-glucose was purchased from Sigma-Aldrich (Shanghai, China). Four different procedures were used to prepare silver nanoparticles (AgNPs) in this study (Figure 1A). AgNPs-1 and AgNPs-2 were produced by adding 1 mL silver nitrate solution (10^–2^ mol/L) dropwise into 9 mL hydroxylamine hydrochloride solution (1.67 × 10^–3^ mol/L) containing 3.33 × 10^–3^ mol/L sodium hydroxide, with a flow velocity of 1 mL/min and 40 mL/min, respectively. AgNPs-3 and AgNPs-4 were produced by adding 1 mL hydroxylamine hydrochloride solution (1.5 × 10^–2^ mol/L) containing 3 × 10^–2^ mol/L sodium hydroxides dropwise into 9 mL silver nitrate solution (1.11 × 10^–3^ mol/L), with a flow velocity of 1 mL/min and 40 mL/min, respectively. The flow velocity was controlled with a syringe pump (Longer, China). After dropwise addition, the solutions were gently mixed immediately. This silver colloid solution is referred to as 1× in the text. The silver colloids were stored at 4°C in the dark for further use.

To evaluate the size and stability of AgNPs, the fresh silver colloids were stored at 4°C in the dark for 48 h, and the UV–Vis spectra and nanopotential were recorded at 0, 4, 8, 24, and 48 h. The UV–Vis spectra of silver colloids were obtained using a UV–Vis spectrometer (UV-1700, Shimadzu, Japan), and the full width at half absorption maximum was calculated to evaluate the size and stability of AgNPs (Leopold and Lendl, 2003). The size of AgNPs was measured using a Zetasizer Nano ZS (Malvern, United Kingdom) nanoparticle size analyzer (Mathioudakis et al., 2020).

### Preparation of Bacterial Cultures

*Escherichia coli* DH5α was purchased from TransGen Biotech (Beijing, China). *Dietzia sp.* DQ12-45-1b (Wang et al., 2011) and *Bacillus subtilis* BSw800 were isolates stored in our laboratory. *E. coli* DH5α and *Bacillus subtilis* BSw800 were cultured in lysogenic broth (LB) medium at 37°C and stirred at 150 rpm. *Dietzia sp.* DQ12-45-1b was cultured in GPY (Glucose, 10 g/L; Peptone, 10 g/L; Yeast extract, 5 g/L) medium at 30°C and stirred at 150 rpm. All bacterial cultures were harvested at the exponential phase, centrifuged at 4°C and 3000 × g for 5 min, and washed three times with PBS (NaCl 0.137 mol/L, KCl 2.7 × 10^–3^ mol/L, Na_2_HPO_4_ 4.3 × 10^–3^ mol/L, KH_2_PO_4_ 1.4 × 10^–3^ mol/L, pH adjusted to 7.2) to remove the medium. The bacterial cells were then suspended in PBS to expected densities for further use.

For analysis of isotopically labeled cells, *E. coli* DH5α was cultured in M9 medium supplied with different proportions of ^13^C_6_-glucose (Kubryk et al., 2015). Cells were harvested at the exponential phase and prepared as described above.

### Preparation of the Membrane Fractions

The inner membrane, outer membrane and cytoplasm were separated as previously described (Marchand et al., 2012) with a small modification. Briefly, *E. coli* DH5α cells were harvested from LB medium at the exponential phase by centrifugation at 4°C and 3000 × *g* for 5 min. Cells were then washed three times with PBS. Then, the cells were broken via bead-beating three times in 5 min intervals on ice, followed by centrifugation at 7500 × *g* for 15 min to remove the intact cells. The supernatants were collected and further ultracentrifuged at 150,000 × *g* for 60 min. After centrifugation, the supernatant was collected as the cytoplasmic sample. The pellets containing membrane fractions were resuspended in deionized water and purified by sucrose density gradient centrifugation (56 to 20% (w/v), 150000 × *g*, 2 h). The bands between 56 and 20% sucrose were collected and layered on a sucrose gradient (56, 53, 50, 47, 44, 41, 38, and 35%). After centrifugation at 200,000 × *g* for 42 h, two bands were detected. The upper and lower bands were collected as the inner and outer membrane fractions, respectively.

### Surface Enhancement Using AgNPs

For enhancement, 2 μL of washed bacterial cells were mixed with 2 mL silver colloid. The mixture was allowed to stand for 15 min or centrifuged at different speeds for 5 min for conjunction of AgNPs and cells before SERS measurement. The morphology of AgNPs on cells was detected using TEM. Samples were fixed with isoprene glycol for 1 h before TEM detection. TEM images of the AgNPs were obtained using a JEM 2010 (JEOL, Munich, Germany) at an accelerating voltage of 200 kV.

To optimize the conditions for SERS enhancement, we chose five factors that might influence the effects of enhancement, including the centrifugal force, the AgNPs preparation method, the AgNPs concentration, the ionic strength of the solution used to suspend cells, and the cell density. Among the above factors, the concentration of silver colloid after preparation was referred to as 1×, and the colloid was diluted with deionized water to 0.5× or concentrated to 2× by centrifugation. The ionic strength of the solution used to suspend cells was adjusted with different NaCl concentrations. An orthogonal experiment, which is an important branch of mathematics statistics to determine how to collect data efficiently and simply (Chen and Ni, 2012), was designed to determine the optimal conditions (Table 1). The signal-to-noise ratio was calculated to estimate the effect of enhancement (Greek et al., 1995).

**TABLE 1:** Factors and levels for orthogonal experiments (3^5^).

**Level**	**Factors**				
	**A**	**B**	**C**	**D**	**E**
	**Cell density (cells/mL)**	**AgNPs**	**NaCl (M)**	**Centrifugal forec (g)**	**Concentration of AgNPs**
1	10^6^	AgNPs-1	0	0	0.5×
2	10^7^	AgNPs-2	0.01	5000	1×
3	10^8^	AgNPs-3	0.04	8000	2×

### SERS Measurements

Before SERS measurement, glass slides were ultrasonically cleaned for 30 min in water, followed by another 30 min ultrasonic cleaning in ethanol. Then, the slides were submerged in 50% methanol solution for 2 h, and 2 mL trimethoxy(propyl) silane and 3 mL 25% ammonia solution were added dropwise, followed by stirring overnight. The slides were washed with ethanol three times and dried before use.

For SERS measurement of single cells, 2 μL AgNPs-cell mixture was added dropwise to a glass slide and air dried. All measurements were performed using a confocal Raman spectrometer (Renishaw, United Kingdom). The measurements were carried out with a 785 nm diode laser, and the laser beam was focused onto the sample with a 40× objective. The laser power was in the range of 0.2 to 6 mW, and the acquisition time was 10 s. The frequency stability and accuracy of the apparatus were checked by recording the Raman spectrum of silicon (520.5 cm^–1^). Three different cells were picked for testing of each sample. Spectra were acquired in a range of 600–1800 cm^–1^. Each Raman spectrum was the average of 15 successive scans obtained. SERS spectra were analyzed with WiRE 4.2 and WiRE Batch Converter software.

For enhancement of different cell fractions, samples were vacuum dried and mixed with 2 mL 1 × AgNPs-2, followed by centrifugation at 8000 × *g* for 5 min. The pellet was suspended in 200 μL supernatant. Two microliters of the suspension were added dropwise to a pretreated glass slide and air dried for further Raman measurement as described above.

### Statistical Analysis

For each sample and test, at least three single cells were measured. Baseline correction and normalization of the spectra were done using the WiRE 4.2 software. Principal Component Analysis (PCA) was performed using Vegan package in R. The ggplot2 and scatterplot3d package in R software were used in our study to visualize the results.

## Results

### Optimizing AgNPs Preparation for SERS

We used four methods to prepare the AgNPs. As shown in Figure 1B, AgNPs-2 prepared by the fast dropwise addition of AgNO_3_ into hydroxylamine-hydrochloride solution had an average diameter of 45 ± 4 nm, which was the smallest diameter observed among the four methods. The average diameter of AgNPs-4, which were prepared by fast dropwise addition of hydroxylamine-hydrochloride into AgNO_3_ solution, was also significantly smaller than that of AgNPs-3 prepared by slow dropwise addition. These results suggest that fast dropwise addition and shortening of the reaction time can reduce the size of AgNPs. To test the stability of the AgNPs, all Ag colloids were stored at 4°C for 48 h, and their diameters and UV–Vis spectra were determined during the 48-h storage time. Compared with other AgNPs, AgNPs-2 exhibited the most stable particle sizes. The diameter of AgNPs-2 ranged from 45 ± 4 to 46 ± 3 nm over 48 h at 4°C (Figure 1B). The UV–Vis spectra of AgNPs-2 were also stable during the 48-h storage time at 4°C (Figure 1C). The results suggest that fast dropwise addition of AgNO_3_ into hydroxylamine-hydrochloride solution was the optimal method to prepare small and stable AgNPs.

**FIGURE 2:**
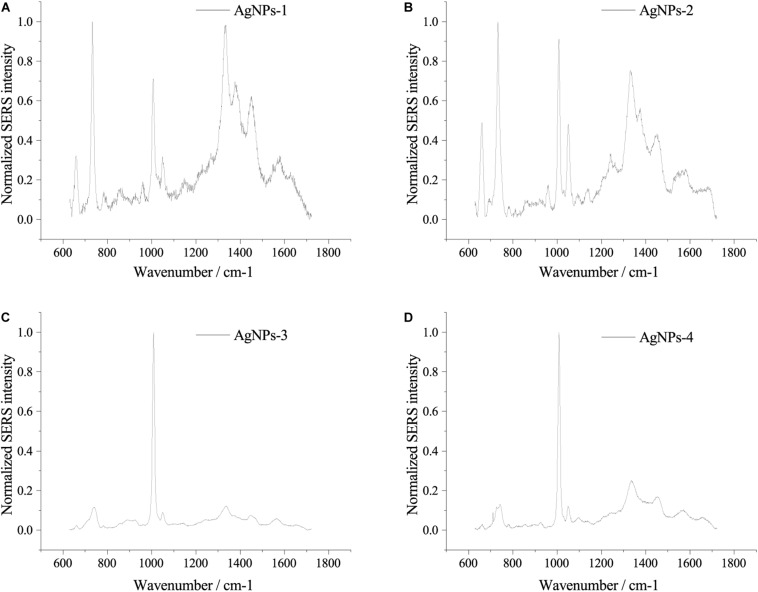
SERS spectra of single *E. coli* cells using different AgNPs. **(A)** AgNPs-1; **(B)** AgNPs-2; **(C)** AgNPs-3; and **(D)** AgNPs-4. For each sample and test, at least three single cells were measured.

Fresh AgNPs prepared using different methods were used to assess their effects on SERS. As shown in Figure 2, AgNPs prepared with different methods showed differences in SERS spectra of single *E. coli* cells. When cells were mixed with AgNPs-1 and AgNPs-2, which were prepared by adding AgNO_3_ dropwise into hydroxylamine-hydrochloride solution, the highest peak in the SERS spectra was at 732 nm^–1^. However, when using AgNPs-3 and AgNPs-4, the highest peak in the SERS spectra was at 1008 nm^–1^. In addition to the shift of the highest peak, when using AgNPs-1 and AgNPs-2, the number of peaks was much higher than when using AgNPs-3 and AgNPs-4. The results indicate that AgNPs generated by adding AgNO_3_ dropwise into hydroxylamine-hydrochloride solution were more favorable for SERS detection. Notably, significant differences were not found between fresh AgNPs-1 and AgNPs-2 or AgNPs-3 and AgNPs-4 (Figure 2), which suggested that the size of the nanoparticles played a weak role in enhancement of SERS signals.

### Effects of Centrifugation on Enhancement of SERS Spectra

**FIGURE 3:**
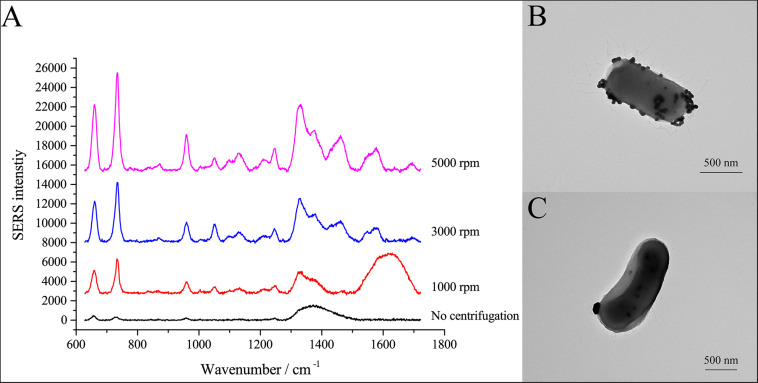
SERS spectra of single *E. coli* cells after centrifugation treatment. **(A)** Comparison of SERS spectra of single *E. coli* cells after centrifugation with different centrifugal forces; **(B)** TEM micrograph of *E. coli* cell with AgNPs after centrifugation at 3000 rpm; **(C)** TEM micrograph of *E. coli* cell with AgNPs without centrifugation.

SERS allows identification of analytes in contact or very close to plasmonic nanostructures (Lombardi and Birke, 2009). Here, we used centrifugation to induce close contact between AgNPs and cells. After the cell pellets were washed with PBS three times, the cells were suspended in PBS and mixed with AgNPs-2. Cells and AgNPs were placed in contact by allowing the mixture to stand for 15 min or by centrifuging the mixture at different speeds. As shown in Figure 3A, different centrifugal rotational force led to large differences in the SERS spectra of single *E. coli* cells. When cells and AgNPs were mixed without centrifugation, only a broad peak at ∼1350 cm^–1^ was observed. After centrifugation, both the average SERS intensity and the number of peaks increased with increasing centrifugal force (Figure 3A). The highest peak in the SERS spectra was at 732 cm^–1^, followed by peaks at 1323 and 654 cm^–1^. To explain the effects of centrifugation on SERS signals, transmission electron microscopy (TEM) was performed. After centrifugation, the surface of the single cell was densely coated with AgNPs (Figure 3B). In contrast, without centrifugation, only a few AgNPs were in contact with the cell surface (Figure 3C). More AgNPs closely attached to the cells might explain the better enhancement after centrifugation.

### Optimal Enhancing Method for SERS

**FIGURE 4:**
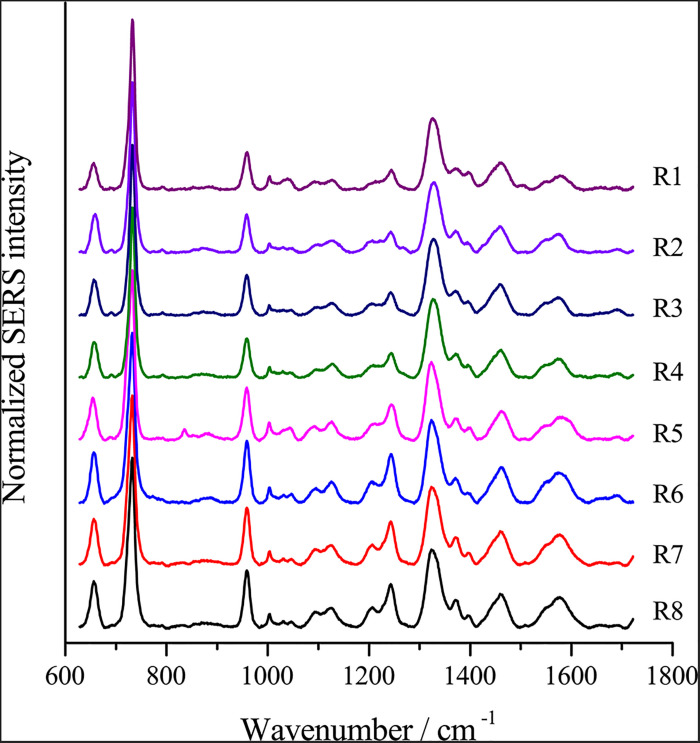
Reproducible single-cell SERS spectra of different *E. coli* cells.

To optimize the enhancement method for SERS, we chose the centrifugal force, method for preparing AgNPs, concentration of AgNPs, ionic strength of the solution used to suspend cells, and cell density as the impact factors, and performed orthogonal experiments. As shown in Table 2, the optimal combination of the factors was A_3_B_2_C_3_D_3_E_1_, under which the highest signal-to-noise ratio would be obtained. To evaluate the reproducibility of the optimal enhancement method, we numbered all cells in the microscopy field and selected cells according to a random number table without bias. All SERS spectra from different single cells showed great similarities (Figure 4). The spectra exhibited characteristic peaks at 654, 732, 958, 1008, 1244, and 1323 cm^–1^. Among all the peaks detected, the relative intensity of the peaks at 1008, 1244, and 1323 cm^–1^ showed minor differences. It has been reported that the 1008 cm^–1^ band of pyridine (ring breathing mode), 1244 cm^–1^ band of C–C stretching mode of lipids and proteins or C–N stretching of proteins, and 1323 cm^–1^ band of guanine may have minor differences at different sites of the cell (Stone et al., 2002; Ruiz-Chica et al., 2004; Stiles et al., 2008), which may cause this minor difference in their Raman spectral peak.

**TABLE 2:** Orthogonal experiment design, results, and analyses.

**Test no.**	**Factors (level)**					Signal-to-noise ratio*
	**Cell density**	**AgNPs**	**NaCl**	**Centrifugal force**	**Concentration of AgNPs**	
1	1	1	1	1	1	7.15 ± 1.96
2	1	2	2	2	2	6.95 ± 1.91
3	1	3	3	3	3	2.24 ± 0.61
4	2	1	1	2	2	10.53 ± 2.44
5	2	2	2	3	3	83.28 ± 4.79
6	2	3	3	1	1	26.06 ± 5.81
7	3	1	2	1	3	18.13 ± 1.08
8	3	2	3	2	1	114.87 ± 25.22
9	3	3	1	3	2	61.25 ± 22.10
10	1	1	3	3	2	1.34 ± 0.29
11	1	2	1	1	3	3.97 ± 1.65
12	1	3	2	2	1	8.86 ± 5.20
13	2	1	2	3	1	30.34 ± 10.37
14	2	2	3	1	2	40.52 ± 8.10
15	2	3	1	2	3	4.78 ± 0.94
16	3	1	3	2	3	58.12 ± 0.41
17	3	2	1	3	1	93.45 ± 10.33
18	3	3	2	1	2	31.25 ± 5.93
K1j	30.51	125.60	181.13	127.07	280.72	
K2j	195.50	343.04	178.80	204.10	151.83	
K3j	377.06	134.43	243.14	271.90	170.51	
k1j	10.17	41.87	60.38	42.36	93.57	
k2j	65.17	114.35	59.60	68.03	50.61	
k3j	125.69	44.81	81.05	90.63	56.84	
Rj	115.52	72.48	20.67	48.28	42.96	

*The summation of signal-to-noise ratios of the peaks at 654, 732, 958, and 1008 nm^–1^. The result shown in Table 2 was mean ± SD after three measurements of each sample.

### The Origins of SERS Spectra of Single *E. coli* Cells

**FIGURE 5:**
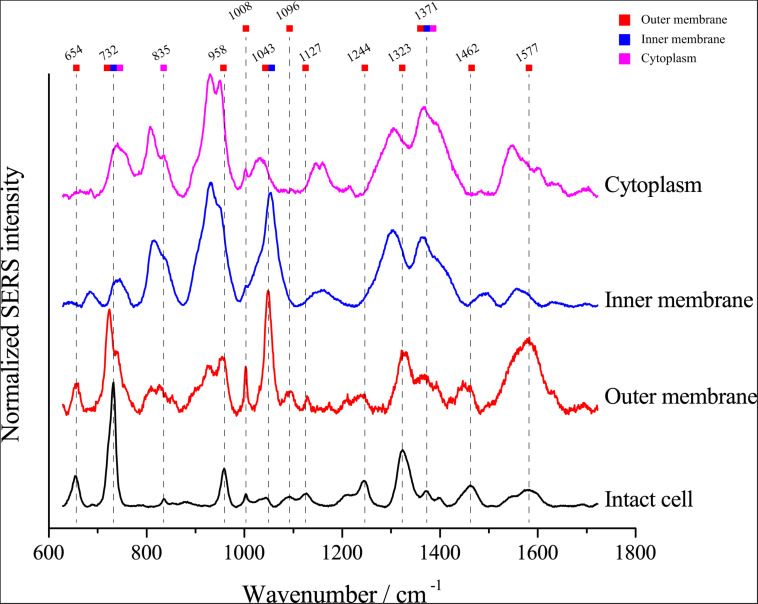
Comparison of the SERS spectra of single *E. coli* cells and different cellular components. The colored squares under the wavenumbers indicate the possible origins of the peaks in the spectrum excited from intact *E. coli* cells.

Van Duyne and Tian, Z.-Q. have proved that the electromagnetic field strength gradually decreased, and the SERS signal strength also gradually decreased when the distance to the surface of nanoparticles was further (Sherry et al., 2006; Tian et al., 2012), we assumed that the SERS signals of single *E. coli* cells might be attributable to the chemical composition of the cell envelope. To investigate whether the SERS spectra arise from the cytoplasm, inner membrane or outer membrane of *E. coli* cells, we separated the cell fractions using ultracentrifugation. Then, we detected the SERS spectrum of each component. As shown in Figure 5, most peaks in the SERS spectra of whole *E. coli* cells were found in the spectra of the outer membrane fraction, including peaks at 654, 732, 958, 1008, 1096, 1127, 1244, 1323, 1371, 1462, and 1577 cm^–1^. The results support the notion that the SERS spectrum of single cells arises from the cell envelope. Notably, although most peaks were found in the outer membrane spectrum, the peaks at 732 and 1323 cm^–1^ were also found in the spectra of both the inner membrane and cytoplasm, suggesting that the corresponding molecules are distributed throughout the cell.

### SERS Spectra of Isotopically Labeled Single *E. coli* Cells

**FIGURE 6:**
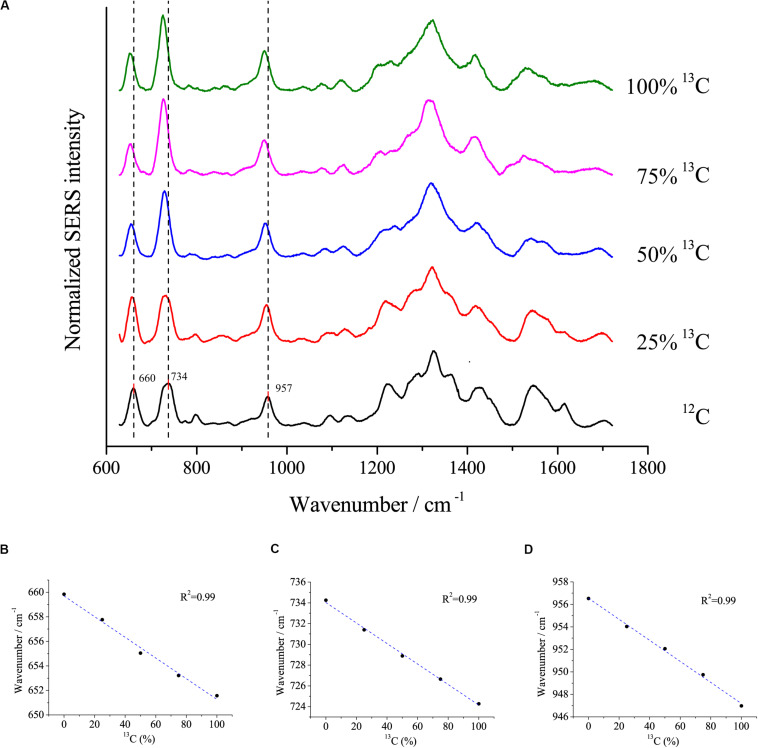
**(A)** SERS spectra of single *E. coli* cells cultivated in different proportions of ^13^C labeled glucose and the linear relationship between the redshifts at **(B)** 660 cm^– 1^, **(C)** 734 cm^– 1^, and **(D)** 957 cm^– 1^ and the proportions of ^13^C labeled glucose.

Surface-enhanced Raman scattering is a popular method to study the uptake of isotopes into cells. Here, we assessed the relationship between redshifts and the proportion of ^13^C incorporation into the biomass of cells using the optimal SERS method. *E. coli DH5*α was cultivated with different proportions of ^13^C_6_-glucose as the sole carbon source. The peaks at 660, 734, and 957 cm^–1^ exhibited clear redshifts of up to ∼6, 10, and 10 cm^–1^, respectively, in cells grown with fully labeled ^13^C glucose compared with cells grown with ^12^C glucose (Figure 6A). Moreover, linear correlations were observed between the redshift of the peaks at 660, 735, and 957 cm^–1^ and the proportion of ^13^C-labeling of the cell (Figure 6B), which suggests that the peaks at these wavenumbers were good indicators to estimate the ratio of isotope incorporation into the cells.

### Discrimination of Bacteria Using SERS

**FIGURE 7:**
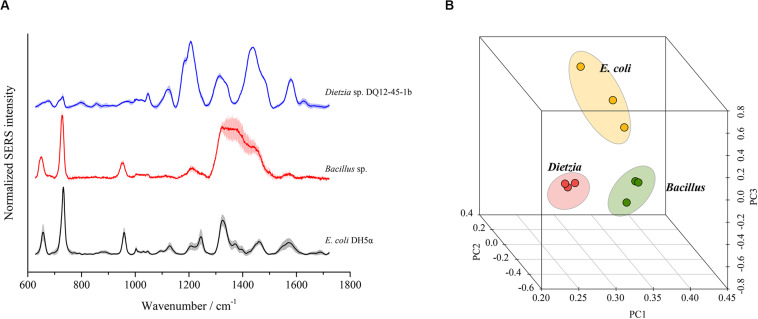
Discrimination of different bacterial species using SERS spectra. **(A)** Characteristic SERS spectra of each species. **(B)** PCA plot based on the SERS spectra of each species.

To test whether SERS using the optimal method could discriminate different bacterial species, we chose three bacterial strains from three distinct phyla, i.e., *E. coli DH5*α from *Proteobacteria*, *Bacillus sp. BSw800* from *Firmicutes*, and *Dietzia sp. DQ12-45-1b* from *Actinobacteria*, for SERS detection. As shown in Figure 7A, each bacterial species presented typical SERS spectra. *E. coli DH5*α and *Bacillus sp. BSw800* showed similar SERS spectra in the region between 600 and 1000 cm^–1^. All three species exhibited distinct spectra between 1100 and 1600 cm^–1^, suggesting significant differences in the biochemical composition of these organisms. PCA showed that the SERS spectra of *E. coli DH5*α, *Bacillus sp. BSw800* and *Dietzia sp. DQ12-45-1b* were grouped into distinct clusters (Figure 7B). We also compared the single-cell SERS spectra of two strains from *Pseudomonas aeruginosa* and *Pseudomonas geniculate* and found the spectra of these two strains were much different (Supplementary Figure S1), which indicated that the optimal SERS method provided sufficient sensitivity to distinguish bacterial species from the same genus. It was notable that the cell growth state would affect the Raman signals greatly. We compared the single-cell SERS spectra of the strain *Dietzia* sp. DQ12-45-1b at different growth states (i.e., the early exponential phase and later exponential phase) and found that the spectra of cells at different stages were different (Supplementary Figure S2A). The results indicated that SERS spectra could distinguish the cells of the same strain at different growth stages. Then we compared the SERS spectra from cells of the same strain at different growth stages with SERS spectra from other strains. Based on the PCA and hierarchical clustering results, we found that SERS spectra could be discriminated by phylogeny firstly, and then by growth stages (Supplementary Figure S2B). Overall, our results suggested that cells from different growth stages would influence the SERS spectra, but they would not influence the discrimination of strains of different species.

## Discussion

In this work, we optimized the conditions for SERS and found that centrifugation and the AgNPs preparation method were crucial for sensitive and reproducible single cell SERS. The origin of the SERS signals was mainly the bacterial outer membrane. The optimized SERS method was used to discriminate bacterial species and indicate the incorporation of isotopes.

Although single cell SERS has been reported for diverse applications in discrimination of bacterial species (Jarvis and Goodacre, 2004; Berus et al., 2020) and detection of pathogens (Boardman et al., 2016), how to improve the reproducibility and sensitivity is still an important issue. The morphology and stability of nanoparticles affects the reproducibility and sensitivity of SERS. A non-homogenous particle size and uncontrollable aggregation process can result in large spectral variations and low reproducibility (Wang P. X. et al., 2016). The shape and size of nanoparticles can be controlled by the reaction conditions (Liu et al., 2013; Zong et al., 2014). We found that for silver colloid preparation, the fast dropwise addition method led to formation of smaller and more stable AgNPs than the slow dropwise addition method, suggesting that a fast, reductive reaction favors better performance of nanoparticles. Based on the Ostwald ripening process, in the fast dropwise addition method, there is not sufficient time for growth and aggregation of the nanoparticles (Bastus et al., 2011; Yang et al., 2014). Comparing the effects of different AgNPs on the enhancement of SERS signals, SERS spectra obtained with fresh AgNPs-1 and AgNPs-2, which were prepared by fast dropwise addition of AgNO_3_ into reductant, exhibited more peaks than those obtained with AgNPs-3 or AgNPs-4. The major difference was that when AgNPs-1 and AgNPs-2 were prepared, the reductant was in excess. The excess reductant might contribute to sufficient reduction of AgNO_3_. Considering the stability and number of peaks, AgNPs prepared by fast dropwise addition of AgNO_3_ into reductant were better for enhancement of SERS signals.

Several methods can be used to coat the cells with nanoparticles in order to form so-called hotspots and obtain ideal enhancement. Among these methods, *in situ* synthesis of silver nanoparticles on bacterial cells (Zhou et al., 2014; Gao et al., 2017) and direct incubation of bacterial cells with metal nanoparticles (Willets, 2009) are frequently used. *In situ* synthesis of Ag colloid ensures the integrity of bacterial cells and generates SERS signals with high sensitivity and specificity. However, the enhancement effect of this method depends on the zeta potential of the cell wall, which is influenced by diverse factors, such as the culture medium (Zhou et al., 2014) and surface structure of distinct bacteria (Wang P. X. et al., 2016). Incubation of cells with AgNPs provides a simple method for enhancement. Although widely used for detection and discrimination of bacterial species (Kahraman et al., 2011; Yang et al., 2016), this method also has disadvantages, such as the long time necessary for incubation before Raman measurement.

The long cell-AgNPs incubation time necessary to obtain ideal enhancement may be due to difficulties in depositing the AgNPs on the cell surface. In SERS, Raman signals of the target molecules are enhanced when they are very close to metal nanoparticles (Panneerselvam et al., 2018). Electromagnetic enhancement and chemical enhancement are thought to be the main mechanisms in SERS (Li et al., 2012b). Electromagnetic enhancement, in which surface plasmon is excited by incident light in the far field and by the Raman scattered oscillating dipole in the near field, predominantly contributes to SERS enhancement. Its effect is restricted in a field less than 5 nm (Panneerselvam et al., 2018). Therefore, shortening the distance between the metal nanoparticles and target molecules is essential for successful enhancement. For single cell enhancement, a strong driving force must be applied to deposit the AgNPs on the bacterial cell surface and form close contact between the cells and AgNPs. In this study, we used centrifugation to drive the deposition of AgNPs. This method is suitable for both Gram-negative bacteria (*E. coli* from *Proteobacteria*), low GC-content Gram-positive bacteria (*Bacillus* from *Firmicutes*) and high GC-content Gram-positive bacteria (*Dietzia* from *Actinobacteria*), which suggests that it may be used generally for detecting diverse bacterial species.

Microbial SERS signals are mainly attributed to flavins (Zeiri et al., 2004), peptidoglycan (Jarvis and Goodacre, 2004), and adenine-derived compounds (Kubryk et al., 2016). The distinct chemical composition of different bacterial species is believed to be the basis of bacterial species discrimination by SERS signals (Hassoun et al., 2017; Genova et al., 2018). However, the correlation between the wavenumber of the peaks and the corresponding compounds is still unclear. Leaking of adenine-derived compounds on the cell surface is thought to be the main origin of the characteristic peak at approximately 730 nm^–1^ in SERS spectra (Kubryk et al., 2016). In this study, we found that the peak at 732 nm^–1^, which is believed to represent compounds such as adenine-derived compounds (Kubryk et al., 2016), could be detected in SERS spectra of the cytoplasm, inner membrane, outer membrane, and intact cells. Surprisingly, the normalized intensity of this peak was higher in spectra of the outer membrane than in those of the cytoplasm, suggesting another possible origin among the outer membrane components. In the SERS spectra of single *E. coli* cells, the major peaks at 654, 958, 1008, and 1323 nm^–1^ were only found in spectra of the outer membrane. These results suggest that the discrimination of bacterial species by SERS is based on the distinct composition of the outer membrane. Further SERS detection of single *E. coli*, *Bacillus*, and *Dietzia* cells also support this point. The SERS spectra of *Bacillus* and *E. coli* shared peaks at 654, 732, and 958 nm^–1^ and showed many similarities. In contrast, the SERS spectra of *Dietzia* were much more different. This result can be explained by the distinct chemical composition of the *Dietzia* cell outer membrane. The cell wall of *Dietzia* and related species differs in composition from other Gram-positive and Gram-negative bacteria (Daffe and Marrakchi, 2019) in that an arabinogalactan layer is covalently linked to the peptidoglycan layer, and a special outer membrane consisting of mycolic acids is present. The distinct composition of the *Dietzia* cell envelope results in SERS spectra that are distinct from those of the other two species.

## Conclusion

In this study, we optimized the enhancement conditions and investigated the possible origins of SERS signals. The results indicated that fast dropwise addition of AgNO_3_ into hydroxylamine-hydrochloride solution was the optimal method to prepare small and stable AgNPs. We also found that fast reaction centrifuging the cell-AgNPs mixture could significantly improve the SERS signals. Finally, the improved method could generate sensitive and reproducible SERS spectra which primarily arose from the outer membrane. Our work provides an improved method for generating sensitive and reproducible SERS spectra.

## Data Availability Statement

The raw data supporting the conclusions of this article will be made available by the authors, without undue reservation.

## Author Contributions

YN and YY designed this study and wrote the manuscript. YY and LA finished the experiments and collected the data. X-LW, Y-QT, and ZX revised the manuscript critically for important intellectual content. All authors read and approved the final manuscript.

## Conflict of Interest

The authors declare that the research was conducted in the absence of any commercial or financial relationships that could be construed as a potential conflict of interest.
