# Supplementary material for: Improvement of Surface-Enhanced Raman Scattering Method for Single Bacterial Cell Analysis
Source: Front Bioeng Biotechnol. 2020 Sep 17;8:573777. doi: 10.3389/fbioe.2020.573777 (PMC7527739; doi:10.3389/fbioe.2020.573777)
Supplement: Supplementary file 1 [file Table_1.DOCX]

Supplementary Material





**Supplementary Figure 1.** Raman spectra of *Pseudomonas aeruginosa* and *Pseudomonas geniculate* strains.


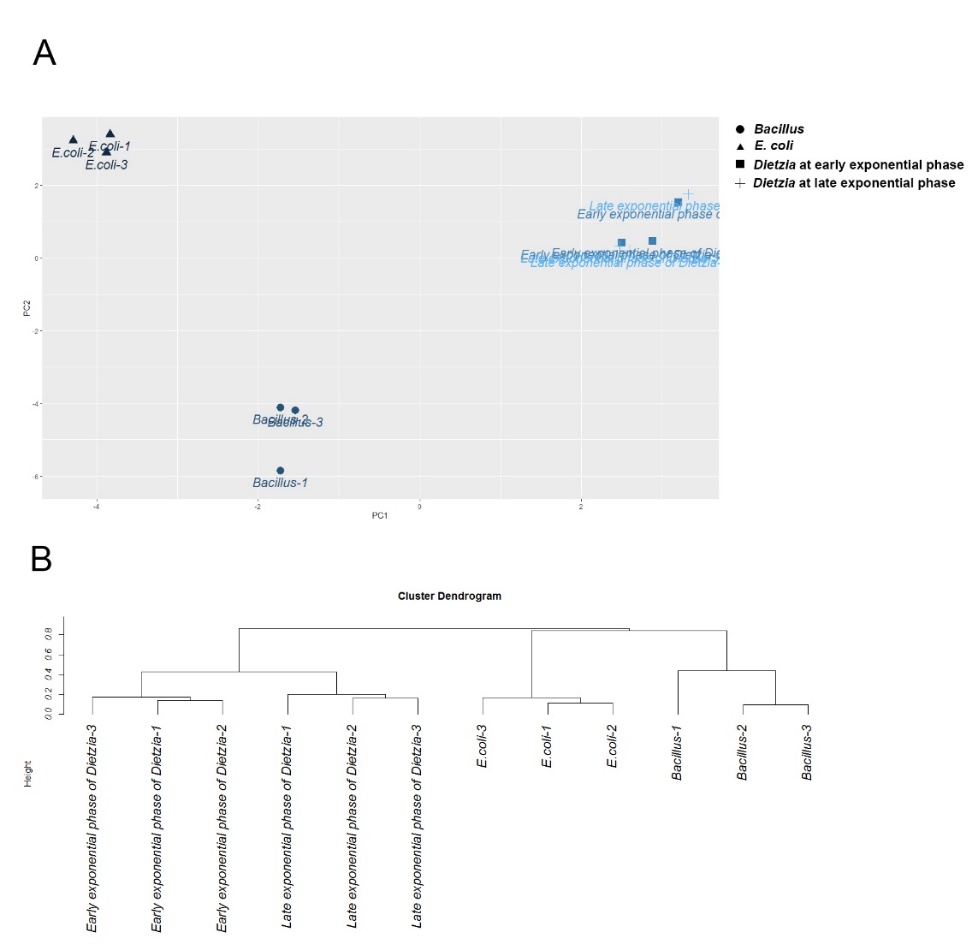


**Supplementary Figure 2.** Discrimination of bacterial cells at different growth states using SERS spectra. (A) PCA plot based on the SERS spectra. (B) hierarchical clustering analysis of SERS spectra of cells at different growth states.
